# Hemorrhagic Pericardial Effusion with Tamponade: A Rare Adverse Effect of Infliximab—Case Report and Literature Review

**DOI:** 10.1155/2016/2576496

**Published:** 2016-10-16

**Authors:** Henry D. Lather, J. Michelle Kahlenberg

**Affiliations:** ^1^University of Michigan Medical School, Ann Arbor, MI, USA; ^2^Department of Internal Medicine, Division of Rheumatology, University of Michigan, Ann Arbor, MI, USA

## Abstract

*Introduction*. Antitumor necrosis factor (TNF) alpha agents are commonly used biologic therapies for a wide variety of rheumatic and inflammatory diseases. Here, we present a case of hemorrhagic pericarditis as a consequence of infliximab and review the literature on pericardial complications stemming from this drug class.* Methods*. For the literature review, search terms using versions of antitumor necrosis factor alpha AND pericardial effusion OR pericarditis OR pleuropericarditis OR cardiac tamponade were used.* Results*. Pericarditis is a rare but serious complication of anti-TNF based therapy, and hemorrhagic fluid is even more rare, with only one additional case reported. It is likely that this complication was secondary to a robust immune response to very high titer anti-infliximab antibodies. Providers should be aware that this complication can occur and that abnormal elevations in procalcitonin may accompany this unusual finding.

## 1. Introduction

Antitumor necrosis factor alpha (anti-TNF*α*) agents are widely used therapies in autoimmune diseases. Here, we present a rare case of hemorrhagic pericardial effusion induced by infliximab and review all reported cases of pericardial effusion during anti-TNF*α* therapy.

## 2. Case Report

We present a 60-year-old man with a history significant for ulcerative colitis requiring total colectomy, primary sclerosing cholangitis requiring liver transplant, chronic renal insufficiency, hypertension, psoriasis, and spondyloarthritis manifested by nearly fused sacroiliac joints, enthesopathies, bursitis, and severe stiffness. He had excellent control of arthritis symptoms on infliximab (5 mg/kg Q6 weeks) but self-discontinued in 2013 for insurance reasons.

**Figure 1:**
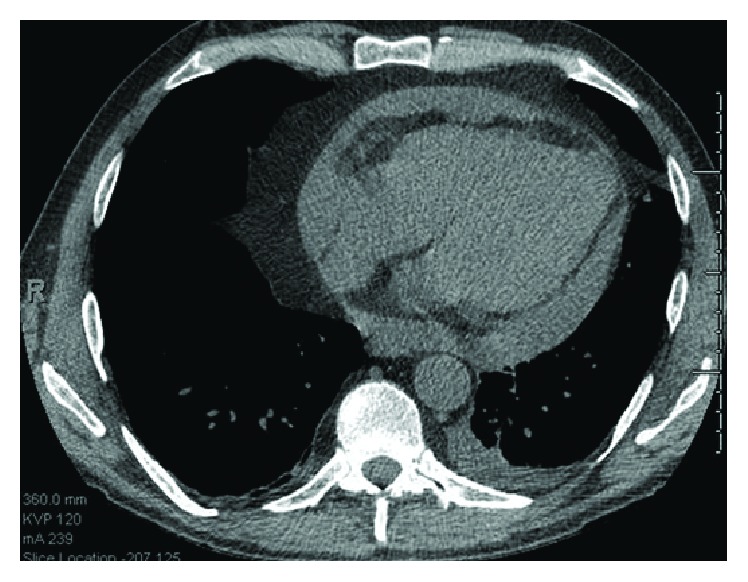
CT of the chest demonstrates a new, moderate, loculated, pericardial effusion and a small left-sided pleural effusion. Right and left atria are normal in diameter.

In July 2014, he received another infliximab infusion secondary to worsening joint pain. One week later, he began to develop shortness of breath, diffuse joint swelling and aches, and general malaise. Nine days after his infusion, he presented to the Emergency Department with severe joint pain in all major joints. He was afebrile, tachycardic (100 s bpm), and hypertensive (140 s–160 s/100 s mmHg). Knee arthrocentesis showed noninflammatory fluid without crystals. Labs were remarkable for white blood cell (WBC) count of 17.1 K/*μ*L (neutrophils 86%), creatinine stable at 2.38 mg/dL, C-reactive protein (CRP) elevated to 4.2 mg/dL (reference < 0.6 mg/dL), total bilirubin elevated to 1.9 mg/dL, and alkaline phosphatase elevated to 208 IU/L. Notably, procalcitonin (PCT) was 118 ng/mL (reference range < 0.05 ng/mL). He was empirically treated with hydration and antibiotics and subsequently began to improve (PCT 56 ng/mL on hospital day (HD) 4). On HD 3, transthoracic echocardiogram (TTE) showed small pericardial effusion and no vegetation. No source of infection was found. Blood, joint fluid, stool, and nasal and urine cultures were negative; urinalysis was unremarkable; viral titers and bacterial serologies were negative; and chest X-ray was unremarkable. Rheumatologic studies were also unremarkable, including C3, C4, ANA, anti-dsDNA, cryoglobulins, and extractable nuclear antigens. He was discharged from the hospital five days after admission and instructed to complete a course of antibiotics. Nine days after discharge, he was again admitted for progressive shortness of breath and recumbent chest pain. Inflammatory labs were elevated as follows: PCT 0.57 ng/mL, CRP 5.3 mg/dL, and Westergren sedimentation rate 82 mm (reference range < 15 mm). TTE following computer tomography on admission showed a moderate-to-large pericardial effusion (Figure 1) with echocardiographic evidence of cardiac tamponade.

Pericardiocentesis was performed and 480 mL of hemorrhagic fluid was drained. Bacterial, tuberculosis, and fungal cultures of pericardial fluid were all negative and no organisms were seen on gram, acid fast bacillus, or fungal stains. Cytology was negative for malignancy. Blood cultures were negative. Anti-histone antibody, rheumatoid factor, and anti-cyclic citrullinated peptide antibody were negative. The patient was started on steroids 60 mg per day, and repeat TTE the next day showed interval decrease in pericardial effusion size. The pericardial drain was pulled the next day and he was discharged home. TTE 2 months after infusion revealed only trace pericardial effusion.

Five weeks after infliximab infusion, anti-infliximab serum antibodies were strongly positive (628,550 ng/mL with reference < 22 ng/mL). Due to absence of documented infection or other identifiable causes of pericardial effusion and in light of highly elevated antibodies against infliximab, it was felt that an immune reaction to anti-TNF*α* therapy was responsible. He was able to taper off high-dose steroids without issue. Two years later, the effusion has not recurred, but he has struggled to manage his joint symptoms without anti-TNF*α* therapy.

## 3. Literature Review

Twenty cases of pericardial complications while on anti-TNF*α* therapy were identified and results are summarized in Table 1.

Thirteen cases of noninfectious pericardial effusion occurred in patients with rheumatoid arthritis (RA), one of which was hemorrhagic [1–7]. It has been suggested that the cause is failure of anti-TNF*α* therapy to control the extra-articular manifestations of RA [3–6], of which pericarditis is common. All 3 cases of infectious pericardial effusion occurred in patients with RA as well [1, 2, 7]. Immunosuppression is likely to have played a role given that patients on anti-TNF*α* therapy are at increased risk of infection [8]. Two of the 4 patients with inflammatory bowel disease (IBD) developed drug-induced lupus erythematosus (DILE), which is thought to have led to pericardial effusion in these patients [9, 10].

Neither RA nor DILE was involved in only 2 cases (including ours). The other case occurred in psoriatic arthritis with adalimumab; however, pericardial fluid was not drained so the fluid type was unknown [11]. Additionally, a case of pericarditis without effusion occurred in a patient with IBD on infliximab [12]. The presumed pathophysiology in all three patients is a hypersensitivity type III immune-complex reaction.

Eight of 20 patients from the literature were known to have continued or restarted the same or different anti-TNF*α* agent without recurrence of effusions within follow-up period. All but one had RA without purulent effusion; he had IBD and noneffusive pericarditis while on infliximab and he was treated with NSAIDs for 2 weeks and at a later time resumed adalimumab with no problems during follow-up [12].

## 4. Discussion

Hemorrhagic pericardial effusion with cardiac tamponade has previously been described in a single case report in a patient with RA receiving the anti-TNF*α* agent adalimumab [5]. Here we present a second patient with hemorrhagic effusion after an infusion of infliximab. Our patient had seronegative arthritis with sacroiliac inflammation, IBD and psoriasis. Although still rare, most patients with pericardial effusion during anti-TNF*α* therapy have RA or DILE whereas ours is only the third reported case of pericarditis without evidence of RA or DILE.

We presume that this complication was secondary to the very high titers of anti-infliximab antibodies present in this patient. This case demonstrates how autoimmune reaction to anti-TNF*α* therapy can initially mimic infection. Our patient presented with tachycardia, leukocytosis, and markedly elevated PCT. Even in autoimmune disease, specificity of PCT (cutoff > 0.5 ng/mL) for bacterial infection is 0.95 in febrile patients [13], but because our patient remained afebrile throughout his initial admission, specificity is unknown. While PCT is known to be slightly elevated in several other noninfectious conditions (e.g., severe organ dysfunction) [14], very high PCT levels in drug hypersensitivity reactions, infusion reactions, and anaphylaxis are rare but do occur, including one case of an infusion reaction against rituximab [15] and one case of anaphylactic shock to risedronate [16].

As illustrated by our case, clinicians should be aware of both noninfectious elevations in PCT and anti-TNF*α*-induced pericardial effusion as rare, but life-threatening adverse effects.

## Figures and Tables

**Table 1:** Anti-TNF*α*-associated pericardial complications (including our case).

Disease	Drug	Pericardial fluid type (number of patients)				
		Infectious	Serous	Hemorrhagic	Unknown	None
RA	Adalimumab	—	2	1	1	—
	Etanercept	2	1	—	5	—
	Infliximab	1	1	—	2	—

IBD	Infliximab	—	—	1	2	1

Psoriatic arthritis	Adalimumab	—	—	—	1	—
